# Successful Use of Subcutaneous Stimulation for Bilateral Sacroiliac Joint Pain

**DOI:** 10.7759/cureus.31495

**Published:** 2022-11-14

**Authors:** Tejas Shah, Ankur Khosla

**Affiliations:** 1 Physical Medicine and Rehabilitation, Hospital of the University of Pennsylvania, Philadelphia, USA; 2 Pain Management, AK Pain, Spine & Neuropathy, Woodlands, USA

**Keywords:** gate control theory, low back pain, peripheral nerve stimulation, spinal cord stimulation, sacroiliac joint dysfunctional pain, neuromodulation

## Abstract

Sacroiliac joint pain (SIJP) has been difficult to properly manage in the medical field. Patients are initially managed with medications and physical therapy but may require further interventions including intra-articular corticosteroid injections, radiofrequency ablation, and sacroiliac joint fusion. Although peripheral nerve stimulation (PNS) and peripheral field nerve stimulation (PFNS) have been used with varying success, subcutaneous spinal cord stimulation (SCS) has not yet been utilized. We present the case of a patient with bilateral SIJP who had successful resolution with the use of subcutaneously-implanted SCS electrode leads.

A 74-year-old female patient with a history of lumbar stenosis status post epidural steroid injection and minimally invasive lumbar decompression presented with year-long chronic low back pain (LBP) with unsuccessful pain relief from medical management and physical therapy. On physical exam, pain elicited with tenderness over both sacroiliac joints with positive Patrick’s and Gaenslen’s test bilaterally. After successful pain relief from a diagnostic SI joint injection, the patient underwent an SCS trial. Trial SCS leads were placed epidurally at T7 and subcutaneously next to bilateral SI joints. Epidural stimulation provided no pain relief after three days. Stimulation was then changed to the subcutaneous leads, with subsequent 90% pain relief. The patient then underwent a permanent implant with subcutaneous lead placement without complications. She reported pain relief ongoing for two years. SIJP is a difficult condition to treat despite various modalities. Recent advances in neuromodulation have shown anecdotal success with PNS. SCS involves electrode leads placed in the epidural space to provide axial back and radicular pain coverage. In this case, however, SCS leads were placed subcutaneously with excellent pain relief. Our case showcases the successful use of subcutaneous-implanted SCS which can provide another viable minimally invasive treatment option in the management of this pain source.

## Introduction

Sacroiliac joint pain (SIJP) remains a confounding issue for medical providers given its unique pathophysiology and location; it is oftentimes confused with low back or hip pain. SIJP may comprise as much as 20% to 40% of all chronic low back pain (LBP) complaints [1-3]. Patients are managed initially with medications and physical therapy but many undergo interventional procedures such as intra-articular injections, radiofrequency ablation, and sacroiliac joint fusion [1,3-4]. Electrical stimulation to either the spinal cord or peripheral nerves has been used for various disease processes and functions to alter pain transmission to the brain. Neuromodulation’s success in SIJP is limited to case reports and observational studies showcasing successful utilization of peripheral nerve stimulation (PNS) and peripheral field nerve stimulation (PFNS), however, the application of spinal cord stimulation (SCS) is much more limited [5-6]. We present the first case of a patient with bilateral SIJP who had successful relief with the use of subcutaneous SCS. 

## Case presentation

A 74-year-old female patient with a history of lumbar stenosis presented with a year-long chronic LBP. Given radicular pain associated with her LBP, the patient underwent multiple interventional procedures including an L4-L5 interlaminar epidural steroid injection and minimally invasive lumbar decompression of the L3-L4 and L4-L5 vertebrae levels. The patient’s LBP and radicular pain persisted despite these procedures and therefore, she underwent the Vertiflex procedure (Vertiflex, Inc, San Clemente, California, USA) of L3-L4 and L4-L5 which improved her radicular symptoms but not her axial back pain. She reported her current LBP as non-radiating and “sharp and burning”; the pain was exacerbated by walking or standing still and improved with sleep and heat application to the area of pain. Her symptoms were accompanied by fatigue and generalized weakness. The patient noted that she would have intermittent shortness of breath, which she attributed to her pain and noted that it only arises when she is exerting herself. During vitals measurement, the patient's oxygen levels saturated above 98% on room air; she was recommended to follow up with her primary care doctor for any further issues should she continue to experience shortness of breath. 

Magnetic resonance imaging (MRI) of the lumbar spine showcased posterior central disc protrusion at L5-S1 and marrow signal alteration in the posterior elements of L3-L4 and L4-L5. She did not have adequate relief with medications including gabapentin, methocarbamol, tramadol or physical therapy. The patient’s vitals were within normal limits. On physical exam, she had decreased sensation on bilateral lower thighs, knees, medial aspect of her lower legs and the dorsum of both of her feet, which she noted has been present for a while. Tenderness to palpation was elicited over both sacroiliac joints. Both Patrick’s and Gaenslen’s test were positive bilaterally. The patient noted a decreased sensation over both lower extremities which has been ongoing since her LBP issues started and was not relieved with the interventions she had undergone.

**Figure 1:**
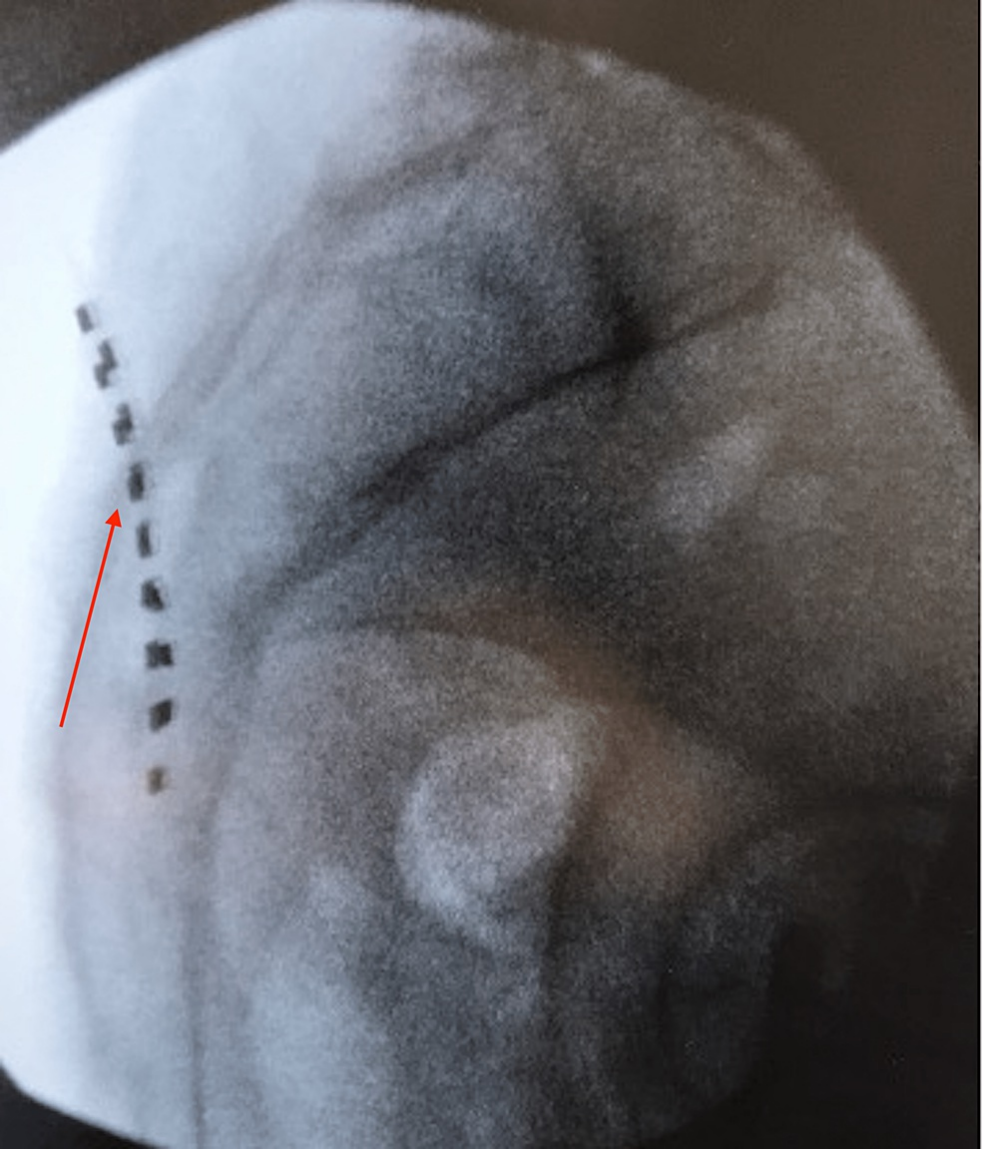
Lateral fluoroscopy view obtained intraoperatively showing spinal cord stimulator octrodes (red arrow) overlying the sacroiliac joint space

**Figure 2:**
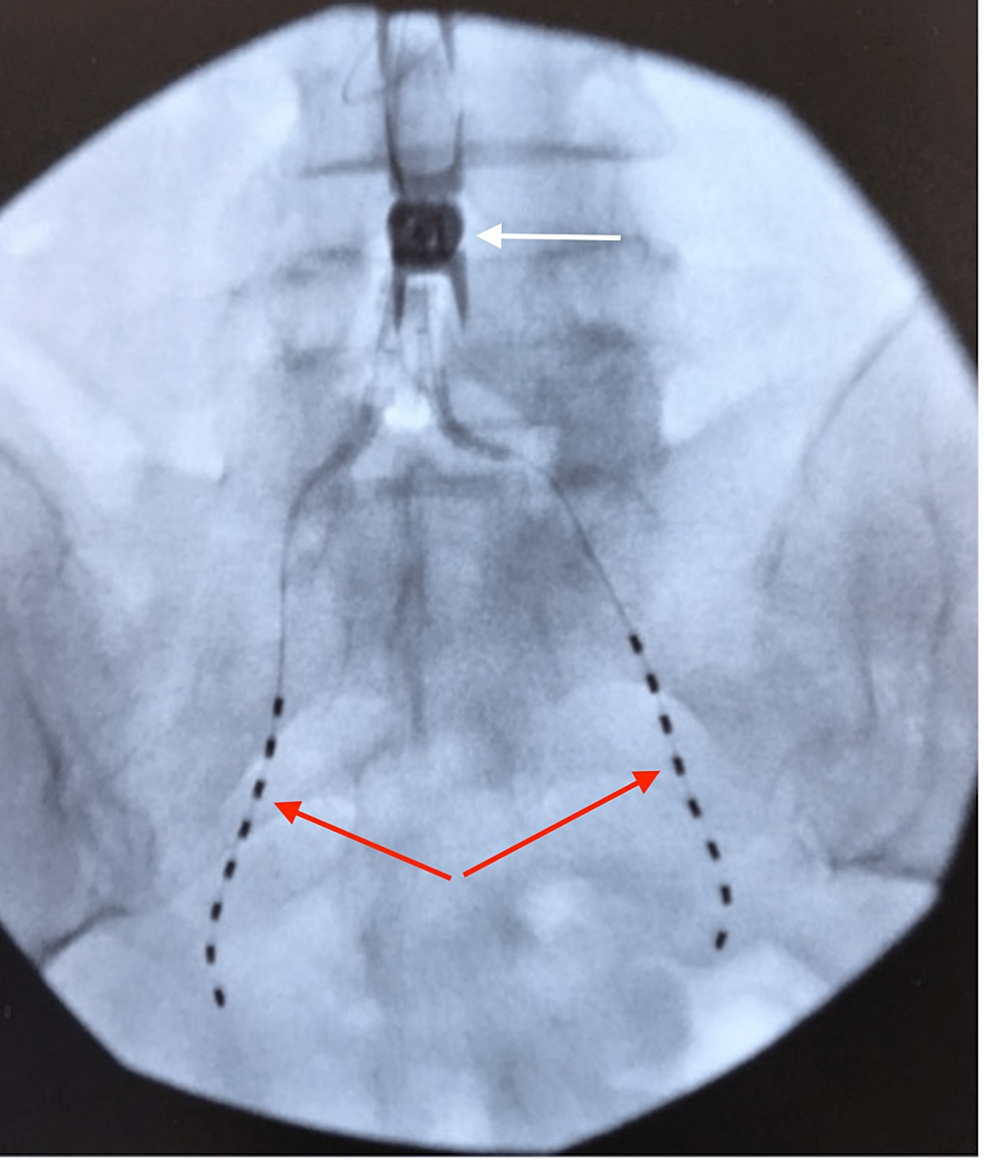
Anterior-posterior fluoroscopy view obtained intraoperatively showcasing vertically aligned spinal cord stimulator octrodes placed in parallel orientation to bilateral sacroiliac joints (red arrows), and Vertiflex interspinous spacer at L4-L5 placed prior to presentation (white arrow)

The patient underwent single intra-articular injections in both sacroiliac joints with successful pain relief, which confirmed her bilateral SIJP. Given the chronicity of LBP with failed interventions, we recommended a spinal cord stimulator (SCS) implant. The patient consented to the burst stimulation SCS trial. Eight contact percutaneous stimulating leads were placed in the epidural space at midline with intra-operative testing confirming paresthesia coverage over painful areas at the top of the T7 vertebral body plane. Simultaneously, Octrode leads (Abbott Medical, Plano, TX, USA) were placed posterior to the sacral plate in the subcutaneous space as far lateral as possible to approximate the sacroiliac joint on both sides, parallel to the sacroiliac joints. On days 1-4, the epidural leads were stimulated with no significant improvement in pain symptoms. On days 5-8, the epidural leads were made inactive while the subcutaneous leads were stimulated with the patient reporting a 90% improvement in symptoms; her visual analog score (VAS) was 1 cm after initially presenting with a VAS of 10 cm. The patient then underwent a permanent implant with only the subcutaneous lead placement without complications (Figures 1-2).

On her two-year follow-up since the procedure, the patient reports continued 90% relief of pain and further noted increased independence with her activities of daily living, including stair negotiation and ambulating without assistive devices.

## Discussion

​​​​​​SIJP was first described by Goldwaite and Osgood in 1905 as an independent contributor to LBP [7]. Although the sacroiliac joint is the largest axial joint in the body, only the anterior third is a “true synovial joint,” representing the union between the ilium and ischium, whereas the posterior aspect is comprised of connecting ligamentous structures [4,7]. The sacroiliac joints aim to provide stability and functions primarily as a shock absorber of the force that is transmitted from the lumbar spine through lateral weight shifting movements [4]. Compared to the lumbar spine, sacroiliac joints can withstand six times the medially-directed force but only 5% of the axial compression load. Therefore, repetitive axial loading and internal or external rotation contributes to the development of SIJP, among other things [1,4].

Patients present with variety of symptoms such as LBP, pain over the buttock, hips, pelvis or groin as well as stiffness [1,2]. SIJP’s clinical symptoms are common with LBP secondary to spinal pathology such as spondylosis or facet arthropathy, making the diagnosis of SIJP more difficult. Our patient underwent multiple interventions including the minimally invasive lumbar decompression and Vertiflex procedures. This may have contributed to the development of SIJP as previous spinal surgery can increase a patient’s risk of developing SIJP up to 43% [8]. In the work-up of SIJP, imaging tools such as MRI help rule out other diagnoses that may mimic this condition [1,3,4]. Although physical exam maneuvers help clue in towards SIJP such as the Patrick's or Gaenselon’s tests, pain relief with a diagnostic fluoroscopy SI joint injection is the gold standard in diagnosing the disease, in line with our patient’s clinical course, making SIJP the likely culprit of presenting symptoms [1,3-4]. 

Innervation of the sacroiliac joints is a well-debated issue in medicine. The majority of sacroiliac joints' innervation comes from the lateral branches of S1 to S3 and medial branches of L4 to L5 but the sacroiliac joints may receive contributions from L2 to S3 nerve roots. These aforementioned nerves serve as the target for radiofrequency ablation (RFA), which aims to denervate the sensory nerve fibers [7]. There however exists a lack of precision in targeting the lateral branches of sacral nerves, due to their variable entrance through the sacral foramen along the lateral border, hence reducing the effect of radiofrequency ablation [5,7]. Furthermore, recent studies show that less patients with SIJP have significant pain relief (greater than 50%) at six months in contrast to three months, which may be explained from the course of natural nerve regeneration but more studies need to be undertaken to further justify this point [7]. In stark contrast to ablating nerves, use of electrical stimulation in SIJP has been on the rise [2-3,5].

PNS and SCS are conjectured to function via the gate control theory of pain where stimulation to the A β fibers blocks transmission from painful C and A δ fibers [5-6,9-10]. This form of neuromodulation aims to modulate cutaneous afferents pain-transmitting nerves in order to inhibit transfer of nociceptive information at the spinal dorsal horn [5,6,9,10]. Specifically, stimulating electrodes are placed proximal to nerves to provide pain relief along the nerve’s dermatomal distribution. PNS has been used successfully for pain relief in occipital, ilioinguinal, trigeminal, and post-herpetic neuralgias [6]. Although limited, there has been use of PNS in SIJP. Guentchev et al., who showcased patients treated with PNS over one year, reported significant decrease in VAS score from 8.8 to 1.6 cm [2]. 

Peripheral nerve field stimulation (PNFS) is a modified version of PNS where leads are placed subcutaneously and unlike PNS, they do not target specific nerves and instead target local nerve endings to provide pain relief [6,9-10]. PNFS has been utilized more often for LBP either as a stand-alone or an adjunct treatment to the standard SCS placed epidurally [10-12]. Although limited in contrast to LBP, PNFS has been studied in the SIJP population. Patil et al. utilized PNFS in 10 patients with treatment-refractory SIJP and over the course of 14 to 29 months; 60% of the patients had significant pain relief, noted as 50% or greater pain relief [6]. Guentchev et al. showed similar success; all 12 patients with treatment-refractory SIJP had at least 50% or greater pain relief over six months, with a notable VAS reduction from 9 to 3.8 cm [5]. A key advantage in using PNFS over SCS or PNS is that it is a safer and simpler procedure for providers in contrast to placing electrodes leads in the epidural space or in close proximity to a large peripheral nerve [9]. Furthermore, the SIJP is not violated with field stimulation, which reduces extensive tissue damage or infection [6]

Although there has been successful utilization of both PNS and PNFS, we report the first successful stand-alone subcutaneous SCS for SIJP. Subcutaneous placement of SCS leads have only been employed frequently as an adjunct to the standard SCS placement and for patients with different disease processes such as chronic LBP or radicular leg pain [12-14]. Hamm-Faber et al. followed 11 patients diagnosed with failed back surgery syndrome (FBSS) for four years after placing either subcutaneous and standard SCS or only subcutaneous SCS with the latter leading to significant pain relief [12]. Similarly, Van Gorp et al. evaluated 97 patents with both chronic leg and back pain [13]. Each patient was randomized to receive either a combination of subcutaneous and standard SCS or only standard SCS [13]. Between the two groups, a higher percentage of patients (42.9%) who received the combination therapy reported at least 50% pain relief [13]. For axial LBP, subcutaneous SCS can help capture the remaining pain coverage, especially in setting of lead migration or hardware complication which commonly leads to loss of efficacy in patients with SCS [12]. Although we present a case of SIJP, it is important to note the common interlinking between this disease and chronic LBP, which are hard to distinguish and commonly co-exist [1]. In fact, as many as 62% of patients diagnosed with only chronic LBP likely have SIJP [9]. Our patient notably has FBSS given her prior history of LBP requiring multiple interventions. Unsuccessful pain relief with the burst SCS during her trial is likely from her lack of radicular symptoms, for which burst stimulation works better [15]. As data has shown success with PNFS, subcutaneous SCS or spinal cord field stimulation may hold potential as an option for patients with SIJP and those with accompanying axial LBP.

## Conclusions

Subcutaneous SCS has only been sparingly used for LBP, however, this is the first case of successful placement of a stand-alone subcutaneous SCS providing ongoing two-year pain relief for SIJP. Future directions should compare subcutaneous and standard SCS as stand-alone or combined options for patients with SIJP. 
